# Postural control during single leg stance in individuals with clinical indication for combined reconstruction of the anterior cruciate and the anterolateral ligaments of the knee: a cross-sectional study

**DOI:** 10.1186/s12891-022-05347-0

**Published:** 2022-04-25

**Authors:** Marilia Novaes, Adriana Carvalho, Juliana F. Sauer, Guilherme C. Brech, Camilo P. Helito, Silvia M. A. João

**Affiliations:** 1 Physical Therapy Service, Instituto de Ortopedia e Traumatologia, Hospital das clínicas HCFMUSP, Faculdade de Medicina, Universidade de São Paulo, 333 Ovidio Pires de Campos St, São Paulo, SP Zip Code: 05403-010 Brazil; 2 Department of Physical Therapy, Speech and Occupational Therapy, Universidade de São Paulo, São Paulo, SP Brazil; 3 Knee Surgery Division, Instituto de Ortopedia e Traumatologia, Hospital das clínicas HCFMUSP, Faculdade de Medicina, Universidade de São Paulo, São Paulo, SP Brazil

**Keywords:** Postural control, Ligament injuries, Knee injuries, Evaluation

## Abstract

**Background:**

Several studies have shown persistent postural control deficits and rotatory instability in patients after isolated Anterior Cruciate Ligament (ACL) reconstruction. There is evidence to support that the Anterolateral Ligament (ALL) plays an important role in the remaining anterolateral rotatory laxity of the knee. There are no further evidences in order to understand how patients with a combined ACL + ALL reconstruction surgery indication behave regarding postural control. The aim of this cross-sectional study was to assess if patients with a clinical indication for the combined ACL + ALL surgery showed a deficient postural control in single leg stance compared to subjects with a regular ACL reconstruction indication and to a control group.

**Methods:**

An assessment of static postural control on single leg stance was performed on a force plate, with eyes open and closed, and the center of pressure (COP) displacement variables were analyzed: maximum and mean amplitude in anteroposterior (AP) and in mediolateral (ML) direction; mean velocity of displacement and area of displacement. Eighty-nine male individuals participated and were divided into 3 groups: ACL Group, ACL + ALL Group and Control Group.

**Results:**

The ACL+ ALL Group showed significantly greater COP displacement in most variables in the injured leg for the eyes closed test, compared to the ACL Group, as detailed: Total ML displacement (9.8 ± 6.77 vs. 13.98 ± 6.64, *p* < 0.001); Mean ML displacement (2.58 ± 2.02 vs. 3.72 ± 1.99, *p* < 0.001); Total AP displacement (9.5 ± 3.97 vs. 11.7 ± 3.66, *p* = 0.001); Mean AP displacement (1.77 ± 0.87 vs. 2.27 ± 0.86, *p* = 0.001); Area of displacement (111.44 ± 127.3 vs. 183.69 ± 131.48, *p* < 0.001).

**Conclusion:**

Subjects with a clinical indication for ACL + ALL combined reconstruction surgery showed increased COP displacement compared to patients with indication for an ACL isolated reconstruction surgery.

## Background

The Anterior Cruciate Ligament (ACL) is one of the main passive knee stabilizers, responsible for restraining anterior and anterolateral tibial translation [1]. Besides its mechanical function, a ligament also provides afferent sensory information about the articular movement and position. This capacity, known as proprioception, along with the motor, vestibular and visual systems, contributes to the ability that the body has to adapt and maintain its segments in a determined position in relation to the environment, referred to as postural control [1–3].

Previous studies have shown that afferent sensory information alterations, possibly caused by ligament disruption and subsequent mechanoreceptor damage, may lead to an altered postural stability, which is the inability to maintain the center of mass inside the base of support (delimited by the feet lateral borders) [4–7]. The body is constantly adjusting to external and internal forces and perturbations, causing postural sways even when we are standing still. We can measure body sway by evaluating the center of pressure (COP), through a force plate. The trajectories and variables obtained from this measure, such as displacement and velocity, are often used to assess postural stability [8–10]. Higher values in these variables usually indicate higher oscillation, and therefore greater postural instability [3].

Several studies have shown that even after ACL reconstruction these postural control deficits, with higher COP oscillation values, may remain [1, 2, 6]. Furthermore, other works have described a persistent rotatory instability in some patients after isolated ACL reconstruction [11–14]. There is evidence to support that the Anterolateral Ligament (ALL) plays an important role in the remaining anterolateral rotatory laxity of the knee, which is seen in up to 25% of the patients undergoing isolated ACL reconstruction. This residual laxity is considered a risk factor for ACL re-rupture [12, 13, 15–18].

Even though there are recent literature showing benefits of the inclusion of an ALL reconstruction in some high-risk populations for ACL reconstruction failure, there is still some controversy among definitive clinical indications for a combined ACL and ALL reconstruction. Nowadays, ALL reconstruction indication is based on some criteria defined in consensus meeting of experts, such as: revision ACL, high-grade pivot shift, young patients (under 20 years of age), excessive anterior tibial translation, participation in pivoting activities, generalized ligament laxity and time since lesion [13, 19, 20]. The finding of an ALL injury on the MRI is not yet considered a criterion for reconstruction indication, according to the latest consensus [19].

Besides those mentioned factors, there have not been any criteria that take into account the patients’ functional deficits in the preoperative period. There is enough evidence to support that an ACL lesion may lead to different degrees of knee dysfunction, in short and long term, such as pain, impaired postural control and muscle strength. However, there is limited knowledge about how such dysfunctions manifest in patients with an ALL reconstruction indication [2, 21]. Ariel de Lima et al. [22] have found, in dissected knees, that the ALL exhibits a peripheral nerve structure, primarily type I and IV mechanoreceptors. This suggests that this ligament also plays a role in the knee proprioception. Its lesion, additional to an ACL injury, could bring even more dysfunction and possibly a postural control impairment [20].

Up to date, studies regarding a postural control evaluation of patients with clinical indication for a combined ACL and ALL reconstruction surgery were not found. Therefore, the main purpose of this study was to investigate the COP displacement and its variables during single leg stance posture of patients with currently accepted clinical indications for combined ACL and ALL reconstruction surgery, and compare the results to those with an isolated ACL reconstruction surgery indication. Our hypothesis was that individuals with clinical indication for the combined surgery would have greater postural stability alteration.

## Methods

### Study design

The ethics committee of our institution approved this cross-sectional study and informed consent was obtained from each participant. The evaluations were performed between January 2018 and December 2019.

The sample size was based on our pilot study. The velocity of COP displacement variable was used, and the mean value of 5.1 +/− 1.7 cm/s was obtained for the single-leg stance test for healthy individuals. We considered as a significant alteration of the variable (which could indicate a dysfunction), any values exceeding 10% the usual coefficient of variation for this variable on the force plate (15%) [23]. Therefore, it was necessary a sample size of at least 28 subjects in each group.

### Participants

Eighty-nine male individuals participated in this study, and were divided into 3 groups. The Control Group consisted of 26 subjects (52 legs tested). Individuals included in this research had no previous history of any neurological, visual, vestibular or musculoskeletal disease, including no previous history of knee injury. Participants were excluded from the study if they were not able to continue the tests due to indisposition.

Patients on our institute waiting list for ACL reconstruction were invited to participate in the study. Sixty-three selected participants presented complete ACL lesion, confirmed by MRI exam, evaluated by a musculoskeletal radiologist and by a knee surgeon, and were divided into 2 groups (according to the knee surgeon and physical therapists evaluation): ACL Group: 30 subjects; ACL + ALL Group: 33 subjects: patients with an ACL lesion and also a pivot-shift test grade III and/or the presence of two of the following criteria: less than 20 years old, time since injury over 1-year, anterior tibial translation difference between legs greater than 7 mm [13, 19]. These criteria were used to define an ALL reconstruction indication since the finding of an ALL injury on the MRI is not yet considered a criterion for reconstruction indication, according to the latest consensus, as stated previously [19].

Patients with meniscal tears were included in the ACL and ACL + ALL Groups, (diagnosed by MRI exam) due to the fact that this injury is frequently associated with an ACL lesion. None of the individuals were professional athletes. Except for the ACL and meniscal injuries in the ACL and ACL + ALL Groups, all groups were free from any neurological, visual, vestibular or other musculoskeletal disease. The absence of pain or swelling was not a requisite for a patient to be included in the study. Participants were excluded from the study if they were not able to continue the tests due to indisposition.

None of the participants received physical therapy treatment after the injury and the International Physical Activity Questionnaire (short version) was used to characterize the subjects’ physical activity level. It was defined as ‘regular physical activity’ those performed by participants within the average of 150–300 min of moderate-intensity, or of 75–150 min of vigorous-intensity physical activity per week [24].

### Experimental set-up

**Fig. 1:**
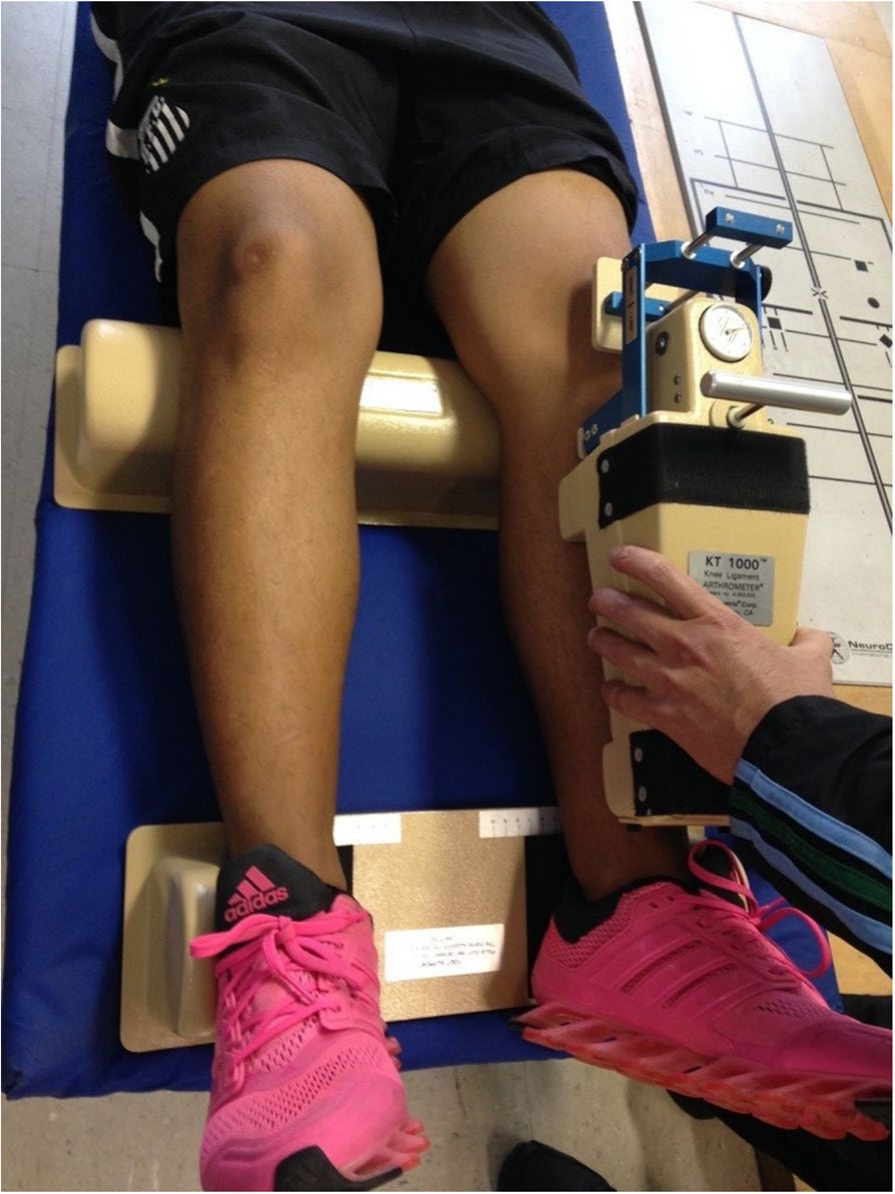
KT-1000 knee arthrometer

Tibial anterior translation was measured by an experienced and trained evaluator with the *KT-1000* knee arthrometer (Fig. 1), following all recommendations from previous studies [25]. The subject stayed in the supine position and a proper bolster (provided with the equipment) was placed under the thighs so that the knees remained at approximately 30° of flexion. A foot support platform was used for maintaining both feet 15 degrees from midline with the hips in external rotation. Before each test, the device was recalibrated to zero, and the anterior tibial translation was performed. The equipment calculated the tibial displacement in mm. The arithmetic average result of three tests for each leg was calculated [26]. This measure was used as one of the criteria for the ACL Groups division.

The postural balance assessment (posturography) was performed on a portable force platform (AccuSwayPlus, AMTI®, MA, USA). For data acquisition, the force platform was connected to a signal-amplifying interface box (PJB-101) that was linked to a computer by means of an RS-232 cable. The data were gathered and stored using Balance Clinic® software, configured to a frequency of 100 Hz with a fourth-order Butterworth filter and a cutoff frequency of 10 Hz. The force platform was used to evaluate semi-static single-leg postures: AccuSwayPlus model, from Advanced Mechanical Technology, AMTI, Watertown, Massachusetts, measuring 50 X 50 cm and 45 mm height, and it is considered a gold standard instrument for this purpose [27].

**Fig. 2:**
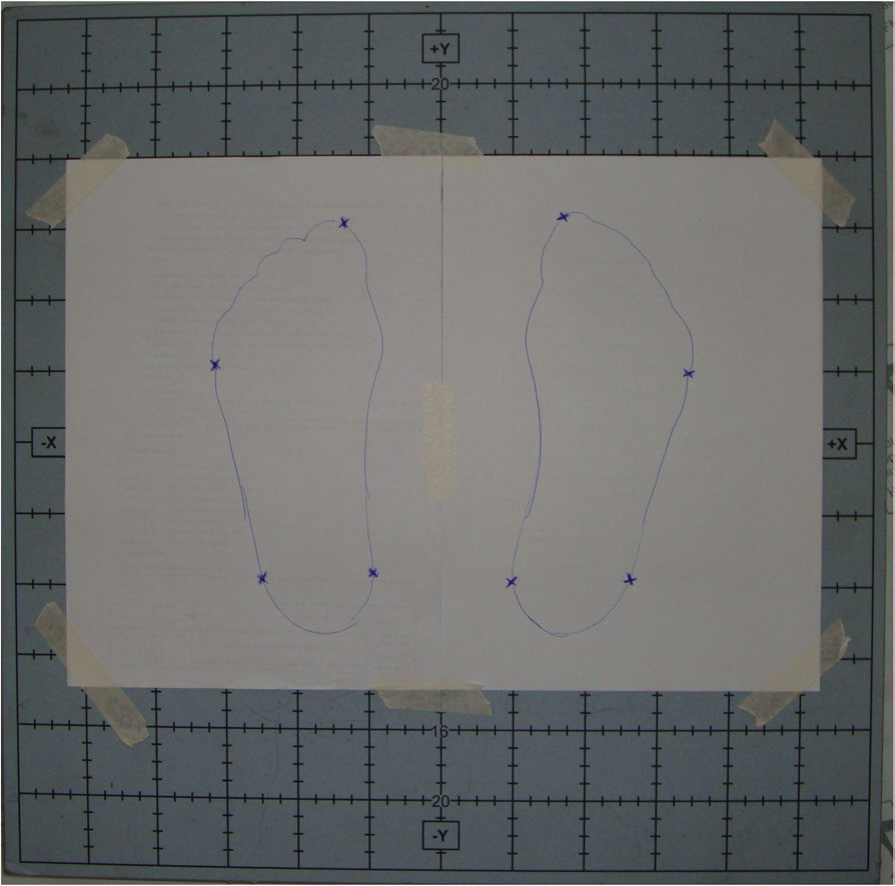
Force platform and feet position

The participants stood barefoot on the platform and were asked to stand straight in a comfortable position, with their feet parallel. They were also instructed to keep their arms along their bodies. The feet positions were recorded on a sheet of paper, and the following points were used as references: hallux distal phalanx, fifth metatarsal head, lateral and medial malleolus (Fig. 2). The evaluator used a proper stick, provided by the manufacturer, and applied a force of approximately 10 lbs. to register the eight points marked on the sheet of paper, so that the program could register the support base and, therefore, calculate the COP displacement based on the coordinates [9].

### Procedure

**Fig. 3:**
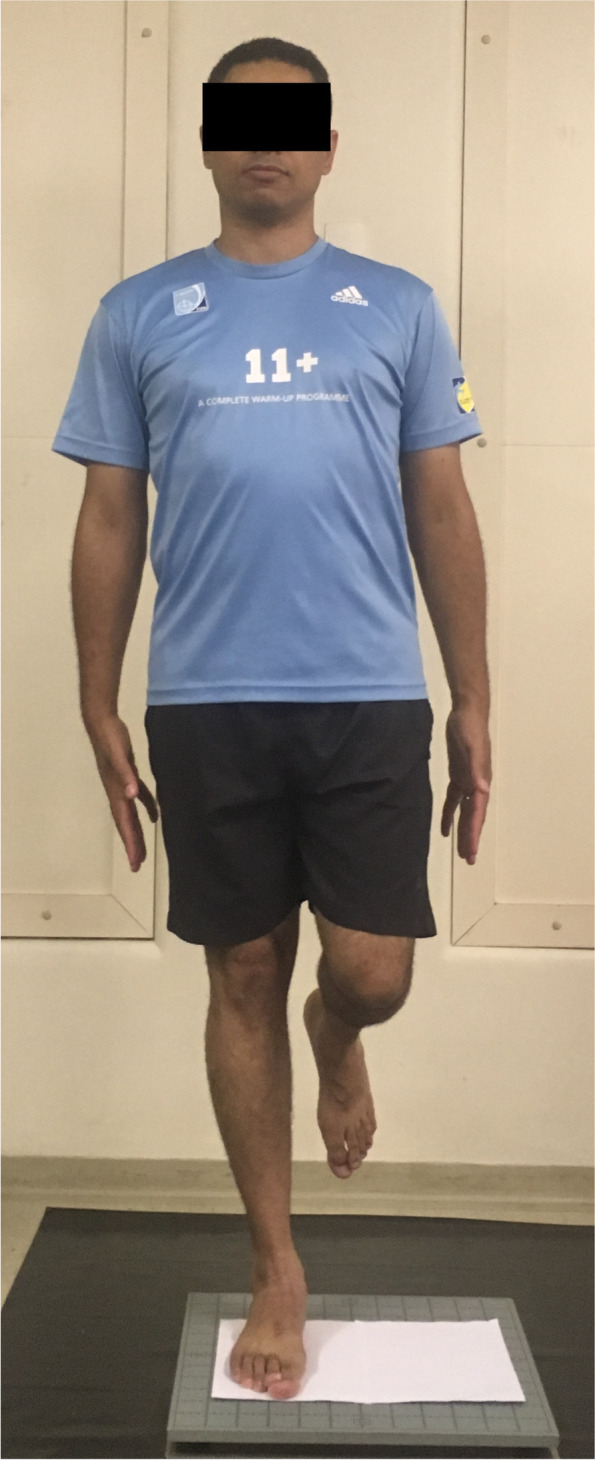
Single-leg stance test

For the single leg stance assessment, the subjects stood on the test limb, at the previous recorded foot position on a sheet of paper, with the knee fully extended and the non-test limb with a 90° knee flexion and hip at a neutral position. Participants were asked to keep their arms relaxed along the body, instructed to fixate on a point about one meter straight ahead of them and maintain their balance (Fig. 3). Participants were allowed to practice once with each leg to get familiarized with the test. Four tests were performed (randomly chosen), all with single-leg stance: (1) injured leg with eyes open, (2) uninjured leg with eyes open; (3) injured leg with eyes closed and (4) uninjured leg with eyes closed. For the eyes closed test, the evaluator stood in front of the participants to make sure they had their closed during the test. For the control group, both right and left legs were tested and the arithmetic average between them was used. The tests with eyes open lasted 30 s and the tests with eyes closed lasted 20 s. It was observed in the pilot study that 30 s was too long even for healthy individuals to maintain balance with eyes closed, so it was decided to make the eyes closed test shorter, also according to literature [8]. There was an interval of 60 s between each test. The arithmetic average result of the three tests for each condition was calculated. If the individual failed performing one of the trials, the average was made out of the remaining attempts.

Dominant leg was not distinguished among individuals based on a previous study showing there is no difference between legs regarding postural control [27].

### Data processing and analysis

The main measure taken into consideration for the study was the center of pressure (COP). The platform measures the three forces (Fx, Fy, Fz) and three moments (Mx, My, Mz) involved in balance, which were used to calculate the COP position and velocity.

For data extraction, the platform was connected to an interface box (PJB-101) for signal amplification, and connected to a computer through a RS-232 cable. Data were collected and stored by the software Balance Clinic®, configured for a 100 Hz frequency, with a high pass Butterworth filter and a cutoff frequency of 10 Hz [9].

The following variables were computed and analyzed: maximum COP amplitude in anteroposterior direction (the sum of the anterior and posterior amplitudes); mean COP displacement in the anteroposterior direction; maximum COP amplitude in the mediolateral direction; mean COP displacement in the mediolateral direction; mean velocity of COP oscillation (calculated from the displacement of the COP in all directions); and area of displacement amplitude, defined as 95% of the area formed by the ellipse of the COP trajectory.

### Statistical analysis

Data were stored in an Excel® spreadsheet for Mac and later imported by SPSS® 25 Software for MAC for statistical data analysis. Continuous data were described by the mean and standard deviation, and were submitted to the Shapiro-Wilk test. Continuous data showed asymmetric distribution, and the Kruskal-Wallis test was used to compare the three groups. For the pair’s comparison, the post hoc Mann-Whitney test with Bonferroni correction was carried. Aiming to evaluate the statistical significance, we adopted a type I error ≤ 0.05.

## Results

Group demographics are reported in Table 1. There was no significant difference between groups for age, weight, time since lesion, number of individuals participating in physical activity and individuals presenting a lateral or medial meniscus tear. There was a statistically significant difference between the ACL + ALL Group and Control Group for height (*p* = 0.03), but it was not a clinically relevant difference, (ACL + ALL had smaller values). The results for the anterior tibial translation, performed with the KT-1000 arthrometer show a significant different between the ACL + ALL Group and the other two groups for the injured leg, and between the ACL and Control Groups.

**Table 1:** Demographic data of enrolled patients

	ACL Group (***n*** = 30)	ACL + ALL Group (***n*** = 33)	Control Group (***n*** = 26)	***P*** Value
**Age (years)**	27.6 ± 8.3	29.8 ± 9.9	28.1 ± 3.5	0.506
**Height (cm)**	176.9 ± 6.7	173.9 ± 6.6†	178.4 ± 8.3	0.03
**Weight (kg)**	82.5 ± 12.9	79.2 ± 12.7	81.8 ± 12.9	0.553
**Physically active** **(number of participants)**	25	20	21	0.07
**MM tear** **(number of participants)**	13	9	0	0.165
**LM tear** **(number of participants)**	3	9	0	0.165
**ATT-Injured leg (mm)**	9.7 ± 2.4†	11.8 ± 2.9†*	3.9 ± 1.5	<0.001
**ATT-Uninjured leg (mm)**	4.3 ± 1.87	3.8 ± 1.53	3.9 ± 1.5	0.478
**Time since lesion** **(months)**	33.56 ± 36	31.58 ± 36.96	0	0,881

*Legend*: *ATT* Anterior tibial translation, *MM* Medial meniscus, *LM* Lateral meniscus. †*p* < 0.05, compared to Control Group. *p < 0.05, compared to the ACL Group

Tables 2 and 3 shows results for all groups regarding the outcome measures: maximum COP amplitude in anteroposterior direction (the sum of the anterior and posterior amplitudes); mean COP displacement in the anteroposterior direction; maximum COP amplitude in the mediolateral direction; mean COP displacement in the mediolateral direction; mean velocity of COP oscillation (calculated from the displacement of the COP in all directions); and area of displacement amplitude, defined as 95% of the area formed by the ellipse of the COP trajectory.

**Table 2:** Single-leg stance test, eyes open, on the injured and uninjured leg

	ACL Group	ACL + ALL Group	Control Group	***P*** Value
**Injured leg**				
TOTAL ML (cm)	3.17 ± 0.91†	3.11 ± 0.69†	2.74 ± 0.44	**0.019**
MEAN ML (cm)	0.6 ± 0.31	0.57 ± 0.13	0.53 ± 0.12	0.159
TOTAL AP (cm)	3.88 ± 0.91	4.5 ± 2.1	3.86 ± 0.78	0.725
MEAN AP (cm)	0.71 ± 0.18	0.82 ± 0.36	0.74 ± 0.2	0.236
VEL (cm/s)	3.59 ± 1.17	4.27 ± 1.54	3.64 ± 0.8	0.216
AREA (cm^2^)	7.44 ± 3.53	8.55 ± 4.44	6.56 ± 1.95	0.082
**Uninjured leg**				
TOTAL ML (cm)	2.86 ± 0.41	3.03 ± 0.64†	2.74 ± 0.44	**0.042**
MEAN ML (cm)	0.53 ± 0.08	0.56 ± 0.14	0.53 ± 0.12	0.441
TOTAL AP (cm)	4.16 ± 1	4.29 ± 1.31	3.86 ± 0.78	0.241
MEAN AP (cm)	0.79 ± 0.19	0.77 ± 0.22	0.74 ± 0.2	0.658
VEL (cm/s)	3.62 ± 0.9	3.91 ± 1.06	3.64 ± 0.8	0.393
AREA (cm^2^)	7.74 ± 2.16	7.27 ± 2.23†	6.56 ± 1.95	**0.040**

*Legend*: The values are presented as mean ± SD. *TOTAL AP* maximum COP amplitude in the anteroposterior direction, *MEAN AP* mean COP displacement in the anteroposterior direction, *TOTAL ML* maximum COP amplitude in the mediolateral direction, *MEAN ML* mean COP displacement in the mediolateral direction, *VEL* mean velocity of oscillation, *AREA* area of displacement amplitude. †*p* < 0.05, compared to Control Group

**Table 3:** Single-leg stance test, eyes closed, on the injured and uninjured leg

	ACL Group	ACL + ALL Group	Control Group	***P*** Value
**Injured leg**				
TOTAL ML (cm)	9.83 ± 6.77†	13.98 ± 6.64†*	6.27 ± 4.28	<0.001
MEAN ML (cm)	2.58 ± 2.02†	3.72 ± 1.99†*	1.48 ± 1.23	<0.001
TOTAL AP (cm)	9.5 ± 3.97	11.7 ± 3.66†*	8.08 ± 2.74	0.001
MEAN AP (cm)	1.77 ± 0.87	2.27 ± 0.86†*	1.41 ± 0.55	0.001
VEL (cm/s)	9.83 ± 3.03	11.32 ± 2.88†	8.74 ± 1.98	0.001
AREA (cm^2^)	111.44 ± 127.3†	183.69 ± 131.48†*	46.95 ± 76.9	<0.001
**Uninjured leg**				
TOTAL ML (cm)	8.39 ± 6.21†	12.02 ± 7.29	6.27 ± 4.28	0.003
MEAN ML (cm)	2.13 ± 1.79†	2.99 ± 2.09†	1.48 ± 1.23	0.017
TOTAL AP (cm)	8.37 ± 2.41	9.37 ± 2.77	8.08 ± 2.74	0.147
MEAN AP (cm)	1.59 ± 0.69	1.97 ± 0.9	1.41 ± 0.55	0.130
VEL (cm/s)	9.23 ± 2.88	10.25 ± 3.63	8.74 ± 1.98	0.174
AREA (cm^2^)	45.6 ± 31.63†	60.51 ± 38.69†	46.95 ± 76.9	0.005

*Legend*: The values are presented as mean ± SD. *TOTAL AP* amplitude in the anteroposterior direction, *MEAN AP* mean displacement of COP in the anteroposterior direction, *TOTAL ML* maximum amplitude in the mediolateral direction, *MEAN ML* mean displacement of COP in the mediolateral direction, *VEL* mean velocity of oscillation, *AREA* area of displacement amplitude. †*p* < 0.05, compared to Control Group. *p < 0.05, compared to the ACL Group

Table 2 shows the results for all groups in single leg stance with eyes open. There was a statistically significant difference in both injured groups (larger values) compared to control group on mediolateral displacement for the injured leg, and also on the non-injured leg of the ACL + ALL Group (larger values) compared to Control Group, for mediolateral displacement and area of displacement.

Table 3 shows the results for single leg stance with eyes closed. There was a statistically significant difference between the ACL Group and Control Group (larger values) for mediolateral maximum amplitude and mean mediolateral displacement on both injured and uninjured legs. For the ACL + ALL Group compared to control group, there was a difference (larger values) between all variables for the injured leg and on mediolateral displacement for the uninjured leg.

There was a statistically significant difference between ACL and ACL + ALL Groups for the injured leg in all variables except for velocity of displacement, meaning greater COP displacement in the ACL + ALL Group.

Table 4 shows the *p*-value for difference between injured and uninjured leg in both groups (ACL Group and ACL + ALL Group). There was a significant difference between legs in the ACL Group for mean anteroposterior COP displacement in the eyes open condition. For the eyes closed test, there was a significant difference between legs in the ACL + ALL Group, for the mean mediolateral, maximum anteroposterior, mean anteroposterior and total area of COP displacement.

**Table 4:** Difference between injured and uninjured leg in the eyes open and eyes closed single leg stance test: p-value

	ACL Group	ACL + ALL Group
**Eyes open**		
TOTAL ML (cm)	0.221	0.581
MEAN ML (cm)	0.428	0.597
TOTAL AP (cm)	0.064	0.981
MEAN AP (cm)	0.011*	0.631
VEL (cm/s)	0.797	0.225
AREA (cm^2^)	0.074	0.517
**Eyes closed**		
TOTAL ML (cm)	0.247	0.949
MEAN ML (cm)	0.258	0.006*
TOTAL AP (cm)	0.159	0.006*
MEAN AP (cm)	0.530	0.031*
VEL (cm/s)	0.360	0.245
AREA (cm^2^)	0.069	0.000*

*Legend*: The values are presented as *p* values. *TOTAL AP* amplitude in the anteroposterior direction, *MEAN AP* mean displacement of COP in the anteroposterior direction, *TOTAL ML* maximum amplitude in the mediolateral direction, *MEAN ML* mean displacement of COP in the mediolateral direction, *VEL* mean velocity of oscillation, *AREA* area of displacement amplitude. *p values <0.05

## Discussion

The current study has shown that patients with a clinical indication for combined ACL + ALL reconstruction surgery decreased postural stability in the single-leg stance test with eyes closed than patients with indication for an isolated ACL reconstruction, and then a control group. For the eyes open condition, there was no difference between the injured groups, and there was a significant difference between both groups with lesion and the control group, showing greater mediolateral displacement and, therefore, decreased postural stability.

There is evidence in literature to support the claim that the worst results for both injured groups in the eyes closed single-leg stance test [28–30]. As stated previously, the visual dependence is probably a compensation for the decrease in sensorimotor afferent information [31–33]. The most interesting finding in our study is the significant difference found between both injured groups. Patients with a clinical indication to an ACL + ALL combined reconstruction surgery showed greater values in all variables when compared to the ACL Group, indicating more instability. As described in Kapreli’s et al. [31], there may be in fact an increased body sway after a ligament injury: the mechanoreceptor damage can lead to decreased and altered afferent information, affecting the entire postural control. As stated before, there is evidence to support the presence of mechanoreceptors in the ALL, suggesting its role in knee proprioception and therefore postural control [20].

Even though meniscal tears could lead to greater postural instability [34], both groups had no significant difference regarding the number of patients who presented such lesion. The same happened concerning the time lapse since injury: both groups had similar values. Therefore, we considered these were not major factors contributing to the group’s results differences.

The results showed greater mediolateral COP oscillation for the groups with ACL lesion compared to the control group in both injured and uninjured leg, especially for the eyes closed test. It should be considered that the mediolateral displacement can usually be related to the maintenance of pelvic balance (lateral tilt) during a single-leg activity [35]. Such patients could have more difficulty in adopting strategies to maintain pelvic alignment during the task than healthy subjects, causing the COP to dislocate more than expected. Such bilateral deficits in the injured groups, compared to the control group, could lead to functional deficits in daily activities, such as squatting or maintaining balance in the single-leg stance phase of waling and climbing stairs. Furthermore, they could affect balance in sports that require single-leg control, such as landing, kicking, and pivoting movements [2].

Previous studies have found a greater COP oscillation in single leg-stance with eyes open in patients with an ACL lesion [4, 7, 30, 36, 37]. According to Negahban’s et al. [7] systematic review, this difference can be observed in at least one variable most of the times. However, three of the studies included in the referred review showed no difference in postural control between healthy and injured subjects [29, 36, 37]. We also found no difference between groups with ACL lesion in the eyes open test. One possible explanation would be that the test is not sensitive enough to detect small possible differences between both injured groups. Another explanation concerns the contribution of the visual system to maintain balance. Okuda et al. [29] stated that the contribution ratio of visual input/postural sway was calculated at 74.46%. Vision, therefore, seems to be an important compensator for the ACL injury in postural sway.

The uninjured leg also showed increased postural sway when compared to the healthy control group for both groups with lesion, especially for the eyes closed condition. Other studies also found bilateral deficit in patients with ACL lesion [7, 28, 32]. It is known that the afferent loss in one member could affect the neuromuscular function and stabilization of the contralateral member [7, 31]. Such formulation is based on the hypothesis that if information coming from one knee is diminished, it can be difficult for the high level sensorimotor system to control both members, once they have distinct sensory properties. To avoid such asymmetry, the central nervous system could reduce the contralateral limb’s performance [7]. Previous research using brain activity imaging have in fact demonstrated that the ACL lesion could cause central nervous system reorganization in spinal and supraspinal levels in patients with long time since injury [31].

Similar to Lehmann’s et al. [3] systematic review and meta-analysis, within-group differences of sway magnitudes were found between the injured and non-injured leg, but only for the ACL + ALL Group, in the eyes closed test. Even though both members showed higher COP oscillations, as stated above, the ACL + ALL Group presented greater sway magnitudes in the injured leg, explaining the statistic difference.

Up until this moment, no other study has investigated postural control during single leg stand in patients with a clinical ALL lesion diagnose. However, our results may contribute, together with studies on other impairments in patients with an ALL lesion, to better understand the clinical situation of these patients. Both in cadaveric and case studies, a greater rotatory instability has been observed in knees with an ALL lesion [38–40]. In some of these studies, there is only a resolution when the ligament is reconstructed. Sobrado et al. [14] demonstrated recently that patients who do not undergo a combined surgery when there is an indication show significantly less favorable outcomes at a minimum follow-up of 2 years after ACL reconstruction, regarding higher re-rupture rates and worse subjective functional scores.

A final remark concerns our belief that the results reached in this study have clinical relevance in the field of physical therapy. Knowing that patients may present poorer postural control in the conditions detailed along this study may instigate professionals to assess this matter and address it during rehabilitation programs in the pre and post-operative period, including proper assessments and exercises aiming to improve their balance and stability.

A limitation of this study has to do with the fact that evaluations were done only in static single-leg stance and prior to the surgery. Based on our results, it would be interesting for further studies to investigate the postural control in dynamic activities, such as pivoting, since the ALL is mostly injured in movements that combine knee flexion, dynamic valgus and tibia internal rotation. Such analysis could probably show an even greater difference between groups, and could possibly be observed in tests with eyes open. Furthermore, as a follow-up, it could be of interest for other studies to find out if postural control improves after the ligament reconstruction, and what could be done along the rehabilitation process to ensure an improvement. Despite the fact that only male individuals have been included in the research, which could be interpreted as a limitation of the study, we would like to point that the decision was based in the features that are intrinsic of female subjects, such as increased quadriceps angle and increased posterior tibial slope, which may predispose them to an ACL injury. Also, there has been found that the ALL is thicker in males than females. Altogether, such differences could lead to diverse results in postural control [41, 42].

## Conclusion

Subjects with a clinical indication for ACL + ALL combined reconstruction surgery show significantly increased COP displacement in the single leg stance test with eyes closed than patients with indication for an ACL isolated reconstruction surgery and than a control group. Based on our results, we suggest more studies to investigate the postural control in dynamic activities, such as pivoting, in order to support our findings.

## Data Availability

The datasets generated and analyzed during the current study are not publicly available due to the fact that the part of the data are still being used as part of another research project, but are available from the corresponding author on reasonable request.

## Abbreviations

LCA: Anterior cruciate ligament
COP: Center of pressure
ALL: Anterolateral ligament
MRI: Magnetic resonance imaging
ATT: Anterior tibial translation
MM: Medial meniscus
LM: Lateral meniscus
AP: Anteroposterior
ML: Mediolateral direction
VEL: Velocity
